# Design, printing optimization, and material testing of a 3D-printed nasal osteotomy task trainer

**DOI:** 10.1186/s41205-023-00185-9

**Published:** 2023-07-13

**Authors:** Lauren Schlegel, Eric Malani, Sara Belko, Ayan Kumar, Eric Barbarite, Howard Krein, Ryan Heffelfinger, Morgan Hutchinson, Robert Pugliese

**Affiliations:** 1 Sidney Kimmel Medical College, Thomas Jefferson University, Philadelphia, PA USA; 2 Health Design Lab, Thomas Jefferson University, Philadelphia, PA USA; 3 Department of Otolaryngology, Thomas Jefferson University, Philadelphia, PA USA; 4 Department of Emergency Medicine, Thomas Jefferson University, Philadelphia, PA USA

**Keywords:** 3D printing, Bone material, Simulation, Medical education, Surgery, Material comparison

## Abstract

**Background:**

For difficult or rare procedures, simulation offers an opportunity to provide education and training. In developing an adequate model to utilize in simulation, 3D printing has emerged as a useful technology to provide detailed, accessible, and high-fidelity models. Nasal osteotomy is an essential step in many rhinoplasty surgeries, yet it can be challenging to perform and difficult to receive adequate exposure to this nuanced portion of the procedure. As it currently stands, there are limited opportunities to practice nasal osteotomy due to the reliance on cadaveric bones, which are expensive, difficult to obtain, and require appropriate facilities and personnel. While previous designs have been developed, these models leave room for improvement in printing efficiency, cost, and material performance. This manuscript aims to describe the methodology for the design of an updated nasal osteotomy training model derived from anatomic data and optimized for printability, usability, and fidelity. Additionally, an analysis of multiple commercially available 3D printing materials and technologies was conducted to determine which offered superior equivalency to bone.

**Methods:**

This model was updated from a first-generation model previously described to include a more usable base and form, reduce irrelevant structures, and optimize geometry for 3D printing, while maintaining the nasal bones with added stabilizers essential for function and fidelity. For the material comparison, this updated model was printed in five materials: Ultimaker Polylactic Acid, 3D Printlife ALGA, 3DXTECH SimuBone, FibreTuff, and FormLabs Durable V2. Facial plastic surgeons tested the models in a blinded, randomized fashion and completed surveys assessing tactile feedback, audio feedback, material limitation, and overall value.

**Results:**

A model optimizing printability while maintaining quality in the area of interest was developed. In the material comparison, SimuBone emerged as the top choice amongst the evaluating physicians in an experience-based subjective comparison to human bone during a simulated osteotomy procedure using the updated model.

**Conclusion:**

The updated midface model that was user-centered, low-cost, and printable was designed. In material testing, Simubone was rated above other materials to have a more realistic feel.

**Supplementary Information:**

The online version contains supplementary material available at 10.1186/s41205-023-00185-9.

## Introduction

The need for practicing procedures outside of the operating room and the role of simulation in filling this gap has been increasingly recognized in today’s paradigm of surgical training. The benefits of surgical simulation are numerous including educational, safety, cost, and outcome-based benefits [1]. Three-dimensional (3D) printing allows for rapid development and production of task trainers to create accessible training opportunities, an ideal complement to the traditional surgical training model.

Rhinoplasty is an extremely common and complex operation requiring both thorough knowledge of anatomy and the technical skills necessary to perform it. It is the most common cosmetic surgery done in the United States with 352,555 having been performed in the year 2020, and more being done for functional purposes, reconstruction after trauma, and revision rhinoplasty [2]. While nasal osteotomy is a cornerstone in this procedure, the step is associated with many potential complications including periorbital edema and ecchymosis, mucosal tears, scar formation, and middle vault collapse causing difficulties breathing [3, 4]. Due to the soft tissue envelope covering the important anatomy and reliance on tactile feedback, this step is difficult to teach and learn.

While previous nasal osteotomy task trainers have been described, in this paper we discuss not only our model, but our approach to designing our task trainer [5]. Literature has shown both high and low fidelity models allow for learning experiences, but high fidelity models may have an increased effect on learning [6]. It is for this reason among others, that the authors tested multiple materials to determine which 3D printing material most closely simulated bone in this anatomic region. Our methods may be applied to the creation of task trainers for other difficult procedural steps. There are multiple 3D printing materials now on the market claiming to simulate bone, but limited literature discussing the fidelity of each material’s performance in specific use cases.

## Objective

The objective of this study was to develop an updated, quickly produced, user-centered, low-cost, and bone-like 3D training model for nasal osteotomy.

## Design

### Image segmentation

**Fig. 1:**
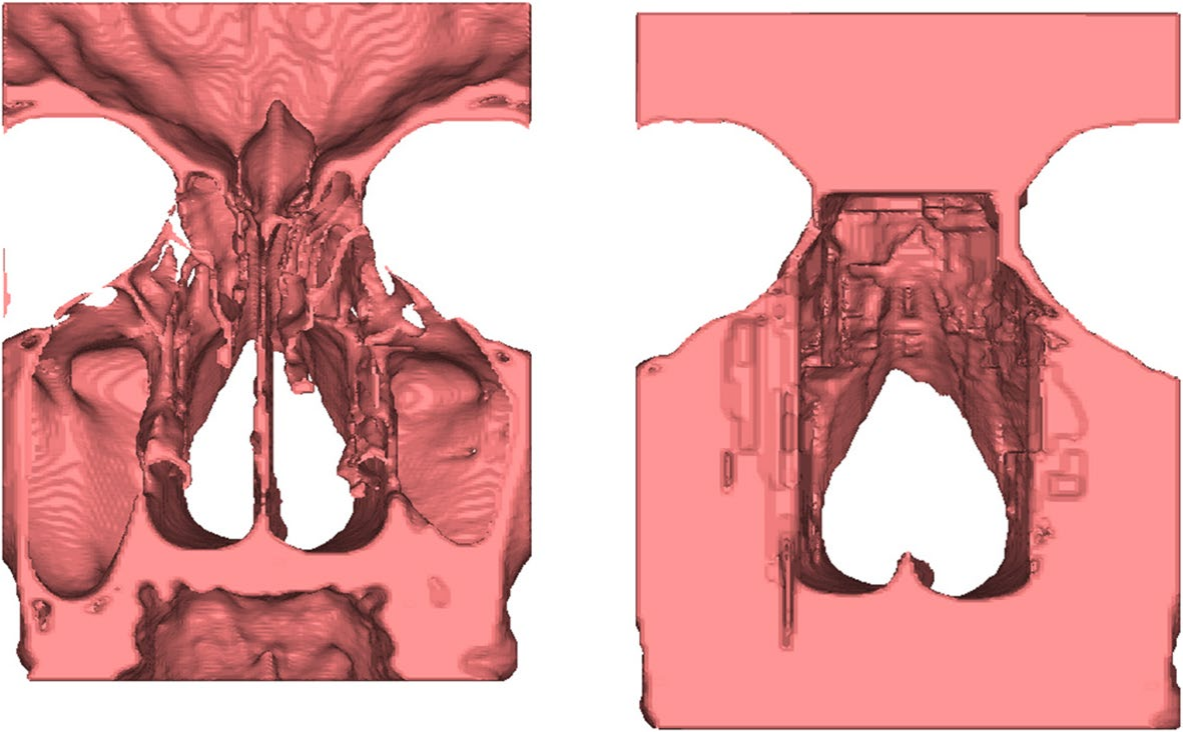
The model was initially segmented in Materialise to use real anatomy as the foundation for the model. This shows the posterior view of the segmented internal facial bone anatomy after **A**) cropping and **B**) removal of small structures and filling in cavities to optimize printability

In the initial round of designing this model, a CT scan ideal for segmenting, with suitable anatomic features, lack of dental artifact, and thin image slices was identified. The imaging had been done using a LightSpeed Pro(16) CT scanner (GE Medical Systems) at 0.625 mm, and approved by three facial plastic surgeons for the use of this model. After deidentification, the information was imported into processing software (Mimics Innovation Suite, Materialise, Belgium). After isolating the bony anatomy from the scan and cropping to focus on the area of interest, all irrelevant internal anatomy was removed (Fig. 1). Additionally, any cavities that would not be utilized for the osteotomies were filled into a solid form decreasing the total print time. The nasal bones were thinned due to feedback that the prior model felt too thick resulting in difficulty performing the osteotomy and smoothed to create a more natural feel.

### Computer aided design

**Fig. 2:**
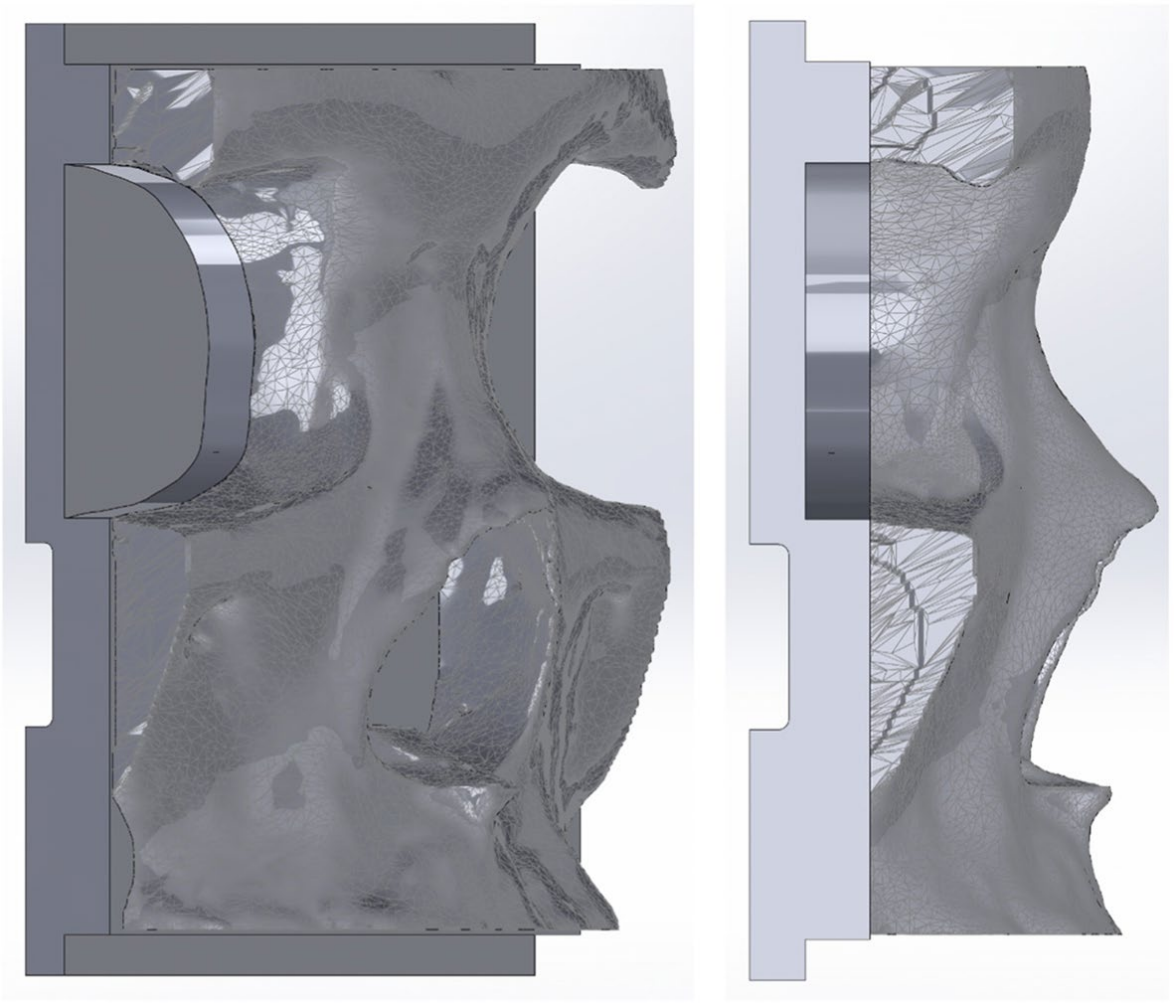
Once the model was segmented, SolidWorks was utilized to further optomize the model. Any areas with unnecessary excess material were reduced, while support pieces were added like a solid base to stabilize the model during the procedure

The STL file was brought into SolidWorks (SolidWorks, Dassault Systèmes, Massachusetts, USA) to finish the design process. To reduce the amount of material needed, the eye sockets were cut back (Fig. 2). Additionally, a base was added to increase stabilization during simulation exercises. A 10 mm thick block was extruded on the posterior side to create a flat base for the model. A section of the back was removed to allow for a strap to secure plastic skin over the model. A 5 mm by 6 mm lip was added on the superior and inferior sides to provide more stability on the table and act as holdfast points (Fig. 2). The eye sockets were cut down 7 mm to decrease material waste and improve print efficiency while retaining anatomic structures needed for performing a nasal osteotomy.

**Fig. 3:**
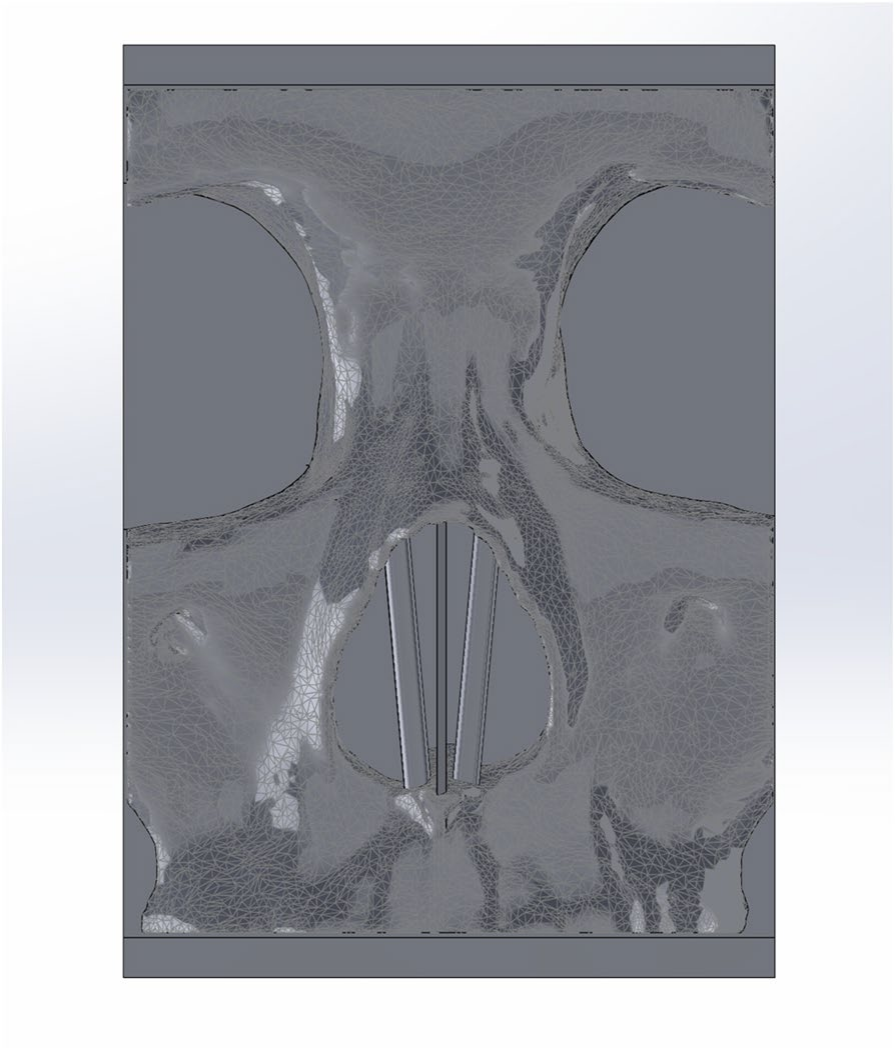
Final nasal osteotomy design with a nasal septum and bilateral support rods added. The base is noted to extend both superiorly and inferiorly to allow easier stabilization during the simulation

A 24 mm diameter semicircle channel was cut midsagittal to improve the post processing dissolution of water soluble PVA support material used and a 1 mm septum was added midsagittal to simulate an anatomic marker and act as a support structure. Bilateral support rods were added to support the nasal bones during osteotomy, replacing the soft tissue that keeps them in place during the real procedure. The final SolidWorks part (Fig. 3) was exported as an STL.

## Printing

To print the various models for testing we utilized two different 3D printing modalities: material extrusion (ME) and vat photopolymerization (VP). While our model optimization led to decreased printing time for the PLA model, post-processing time remained around 12 hours for the ME models as PVA material was used as supports and dissolve time remained a rate limiting step. The dissolve time can be expedited by physically removing PVA supports before and during dissolution. ME was used for printing polylactic acid (PLA), FibreTuff, SimuBone, and ALGA. Ultimaker Cura V5.1.1 was used for slicing and an Ultimaker S5 for printing. The nasal osteotomy model made from Durable V2 resin was printed on a FormLabs Form 3B printer with slicing done in PreForm. The same STL file was used to prepare all models and the appropriate material profiles were imported from Ultimaker’s marketplace into Cura when necessary. The infill for ME models was set to 20% to minimize material usage in the base since the nasal bone area would be solid regardless of infill due to its thin wall thickness. Notably, due to the high bed temperature necessary for printing FibreTuff, the Ultimaker PVA supports were not adhering properly to the build plate. The temperature was decreased a few degrees for the first layer and returned to the profile-specified temperature for subsequent layers. All modifications to the profiles as well as cost and material usage are detailed in Additional file 1. The default layer height for each profile was used and was within a 0.1mm difference. For ME models, once the prints finished, the models were removed from the build plate, the adhesion brim was removed, and models were placed in a four-liter container of warm tap water with a stir bar for approximately 12 hours to allow PVA supports to dissolve. For the VP models, parts were removed from the build plate, washed, and cured according to material specifications, and then supports were manually removed.

## Material testing

### Methods

All five materials were tested at random by board certified facial plastic surgeons and a facial plastic fellow blinded to each other’s preferences. After practicing both percutaneous nasal osteotomy and endonasal osteotomy on a model using standard osteotomes and mallets, each tester filled out a survey specific to their experience with that material. They were blinded to the type and cost of material, in addition to any printing details. Questions about overall value and reality of the models were asked (Additional file 2). Survey responses were exported from Google Forms to Google Sheets (Google, California, USA) and statistics were calculated in Microsoft Excel (Microsoft, Washington, USA).

## Results

After some optimization in printing, each material was successfully printed with the parameters outlined in the methods section. Print time ranged from 478 minutes to 730 minutes and cost ranged from $4.54 - $50.37 depending on the material (Table 1).

**Table 1:** Comparison of the time to print, quantities of materials used, and cost of materials for all five materials tested with the nasal osteotomy model

	**Material**				
	**PLA**	**FibreTuff**	**SimuBone**	**ALGA**	**Durable V2**
**Time to print (per 1 model)**	478 minutes	730 minutes	655 minutes	638 minutes	475 minutes
**Material used**	66.0g	63.9g	75.7g	70.7g	155mL
**Support (PVA) used**	17.5g	15.4g	15.5g	15.5g	N/A
**Cost per gram material**^a^	$0.07	$0.51	$0.13	$0.03	$0.42
**Cost per gram support (PVA)**^a^	$0.13	$0.13	$0.13	$0.13	N/A
**Total material cost**^a^	$6.95	$34.87	$11.96	$4.54	$50.37

^a^These costs are reflective of current local costs in the United States of America

Two attending facial plastic surgeons and one fellow facial plastic surgeon tested the materials and filled out the survey. Performance of the materials in tactile feedback, audio feedback, and material limitation varied greatly between each material and at times depending on the technique used (Table 2). SimuBone’s ratings were best across all categories for both techniques. All the other materials performed well in some categories and not well in others (Tables 2 and 3). While the Durable V2 resin material scored very well when utilized for the endonasal technique, it did not perform nearly as well during the percutaneous portion of testing (Table 2).

**Table 2:** Survey results from evaluations of material performance in nasal osteotomy rating each category out of five (1: very poor, 2: poor, 3: neutral, 4: good, 5: very good) reported as averages of the ratings of the three facial plastic surgeons

	**Endonasal**	**Percutaneous**
**Tactile Feedback**		
*FibreTuff*	2.3	2.7
*Alga*	3.3	3.3
*Durable V2 resin*	4.3	2.7
*PLA*	3.0	3.0
*SimuBone*	4.7	4.0
**Audio Feedback**		
*FibreTuff*	2.3	2.3
*Alga*	3.7	3.7
*Durable V2 resin*	3.3	2.7
*PLA*	2.7	3.0
*SimuBone*	4.3	4.7
**Material Limitation**		
*FibreTuff*	2.0	1.7
*Alga*	2.7	3.3
*Durable V2 resin*	3.0	1.7
*PLA*	2.3	2.7
*SimuBone*	4.3	4.0

**Table 3:** Each material was rated for overall value and overall reality during the material testing on a 5-point scale (1: very poor, 2: poor, 3: neutral, 4: good, 5: very good) and the scores were averaged

	**Overall Value**	**Overall Reality**
*FibreTuff*	1.7	1.7
*Alga*	3.3	3.3
*Durable V2 resin*	3.7	3.0
*PLA*	3.7	2.3
*SimuBone*	4.7	4.3

SimuBone was chosen as the first material of preference by all 3 surgeons (Table 4). For second preference, there was more variability in responses with Alga, Durable V2 resin, and PLA each earning second preference by one of the surgeons. FibreTuff was the least preferred with two surgeons choosing it as their last choice and one putting it in fourth place.

**Table 4:** Facial plastic surgeons ranked the five materials in order of preference after testing

*Material*	**1**^**st**^** Choice**	**2**^**nd**^** Choice**	**3**^**rd**^** Choice**	**4**^**th**^** Choice**	**5**^**th**^** Choice**
*FibreTuff*				1	2
*Alga*		1		2	
*Durable V2 resin*		1	2		
*PLA*		1	1		1
*SimuBone*	3				

## Discussion

The objective of this study was to create a low cost, high fidelity, and time-efficient for production nasal osteotomy model while testing for the superior, 3D printable, bone-like substitute for cadaveric bone in this procedure. Rhinoplasty is an extremely common procedure and therefore all trainees should ideally graduate residency feeling competent in all of the steps. Yet technical training remains a known challenge with 28% of senior residents and 87% of junior residents feeling insufficiently exposed to rhinoplasty during residency with nasal osteotomy to be the technique they were least confident in performing [7]. This has led to a call for focused, maneuver-specific simulation. Preliminary data from this model has been shown to increase confidence, but further studies must be done [8].

In this study, we used an Ultimaker S5 3D printer with dual extrusion capability and a FormLabs Form 3B, relatively affordable and accessible printers. We chose to utilize the software that the authors were most comfortable with; however there are many free software programs that accomplish similar functions. This model was made for the purpose of simulation and therefore is not personalized nor a surgical guide [9]. All of the materials chosen were commercially available and sourced online. No financial support was obtained from the manufacturers for the purchase of these materials, and they were each purchased via standard publicly available purchasing pathways.

We focused on making our model efficient for 3D printing, resulting in a 52.7% reduction in time printing the new model in PLA compared to the previously published model printed in PLA [6]. Although the updated model took less time to print in PLA, the necessary anatomical structures for the osteotomy procedure were kept intact. Additionally, a channel was added to the design when it was observed that it was difficult to remove all the PVA from the area of interest. Although this increased print time in this material, the channel allowed for water to circulate through the model thus making clearing the PVA away from the region of interest easier.

While many materials are listed as mimicking bone, there is a paucity of literature comparing them or describing their ideal use. This study compares the details of their printing and the performance of the materials when used to print training models for nasal osteotomy. We believe this kind of comparison can be useful for others determining the best material for any medical education simulation project involving bone.

This model was designed to be a simpler version of the previously published design; however, it was still printed with a dual extrusion printer using PVA for soluble support. This limits the number of end users that are able to print the model as most commercially available ME 3D printers use a single extruder. Additionally, a major limitation in the material analysis was the small sample size of three experienced surgeons to conduct testing. Proper evaluation of the tactile and auditory feedback of this device requires surgeons to have performed numerous nasal osteotomies on actual patients which limits the number of people qualified to evaluate the models.

A single preliminary test of printing the unaltered model using PLA material without any PVA support on an Ultimaker S5 printer demonstrated that the model is able to be printed with minimal deformity that should not affect functionality. We elected to utilize supports for optimal quality in this material comparison study and further testing is necessary to evaluate the functional quality of the model printed without support. In the future, we hope to do a full resident training conference day with the SimuBone material to explore the impact that may have on the educational experience.

## Conclusion

In this study, we demonstrate the optimization of a previously published nasal osteotomy task trainer using techniques that optimized the design for 3D printing. By creating a model prioritizing printing efficiency and total cost, this new task trainer is more accessible for utilization in simulation of nasal osteotomy in rhinoplasty surgery. Moreover, SimuBone proved to be a more realistic material for simulating the anatomic region of the nasal bone according to our measurement scheme while maintaining accessibility at a relatively low-cost when compared to cadaveric bone. For those with access to a 3D printer and readily accessible supplies, this work allows for high-fidelity and low-cost accessible surgical trainee education.

### Supplementary Information

**Additional file 1: ** Printing profiles for each material. 

**Additional file 2:** Material testing questionnaire for nasal osteotomy simulation.

## Data Availability

Data is available by reasonable request to the corresponding author.

## Abbreviations

3D: Three-dimensional
ME: Material Extrusion
VP: Vat Photopolymerization
PLA: Polylactic acid
