# Correction to: Design, printing optimization, and material testing of a 3D-printed nasal osteotomy task trainer

**DOI:** 10.1186/s41205-023-00188-6

**Published:** 2023-08-21

**Authors:** Lauren Schlegel, Eric Malani, Sara Belko, Ayan Kumar, Eric Barbarite, Howard Krein, Ryan Hefelfnger, Morgan Hutchinson, Robert Pugliese

**Affiliations:** 1 Sidney Kimmel Medical College, Thomas Jefferson University, Philadelphia, PA USA; 2 Health Design Lab, Thomas Jefferson University, Philadelphia, PA USA; 3 Department of Otolaryngology, Thomas Jefferson University, Philadelphia, PA USA; 4 Department of Emergency Medicine, Thomas Jefferson University, Philadelphia, PA USA

**Correction to: 3D Printing in Medicine (2023) 9:20**

10.1186/s41205-023-00185-9

Following publication of the original article [1], the author noticed that in Tables 2, 3 and 4, “Durable V2” was listed as “Flex 80A”. The correct material to be listed has been corrected to “Durable V2”.

The original article [1] has been corrected.
