# Supplementary material for: Design, printing optimization, and material testing of a 3D-printed nasal osteotomy task trainer
Source: 3D Print Med. 2023 Jul 13;9:20. doi: 10.1186/s41205-023-00185-9 (PMC10339601; doi:10.1186/s41205-023-00185-9)
Supplement: Supplementary file 1 — Additional file 1: Printing profiles for each material. [file 41205_2023_185_MOESM1_ESM.docx]

| **Material** | **PLA** | **FibreTuff** | **Simubone** | **ALGA** | **Durable V2** |
| --- | --- | --- | --- | --- | --- |
| **Printer** | Ultimaker S5 | Ultimaker S5 | Ultimaker S5 | Ultimaker S5 | FormLabs Form 3B |
| **Build Plate** | Textured Steel | Textured Steel | Textured Steel | Textured Steel | Standard |
| **Drying** | None | 65°C for 4 hours | 65°C for 4 hours | None | None |
| **Model File** | NO 1 | NO 1 | NO 1 | NO 1 | NO 1 |
| **Slicing Software** | Ultimaker Cura Cura 5.1.1 | Ultimaker Cura Cura 5.1.1 | Ultimaker Cura Cura 5.1.1 | Ultimaker Cura Cura 5.1.1 | PreForm 3.26.2 |
| **Infill** | 20% | 20% | 20% | 20% | 100% |
| **Orientation** | Flat (Horizontal, centered on plate) | Flat (Horizontal, centered on plate) | Flat (Horizontal, centered on plate) | Flat (Horizontal, centered on plate) | Raft (Horizontal, centered on plate) |
| **Print Cores** | AA & BB 0.4mm | AA & BB 0.4mm | AA & BB 0.4mm | AA & BB 0.4mm | N/A |
| **Layer Height** | 0.2mm | 0.15mm | 0.15mm | 0.2mm | 0.1 mm |
| **Wall Thickness** | 0.8mm | 0.8mm | 0.8mm | 0.8mm | N/A |
| **Profile Type** | Default | FibreTuff (from Ultimaker Marketplace) | SimuBone (from Ultimaker Marketplace) | ALGA (from Ultimaker Marketplace) | Default |
| **Resolution** | Fast | Normal | Normal | Normal | Normal |
| **Modifications to profile** | Support = Extruder 2 | Support = Extruder 2  (Extruded 2) Enable Prime Blob = on  Enable Prime Tower = off  Build Plate Temperature = 80ºC  Build Plate Temperature Initial Layer = 70ºC  (Extruder 2) Initial Layer Speed = 10mm/s | Support = Extruder 2 | Support = Extruder 2 | None |
| **Post Processing** | Submerged in lukewarm tap water with agitation for 24 hours or until PVA supports had dissolved completely. Model then left to dry at room temperature for an additional 12 hours. | Submerged in lukewarm tap water with agitation for 24 hours or until PVA supports had dissolved completely. Model then left to dry at room temperature for an additional 12 hours. | Submerged in lukewarm tap water with agitation for 24 hours or until PVA supports had dissolved completely. Model then left to dry at room temperature for an additional 12 hours. | Submerged in lukewarm tap water with agitation for 24 hours or until PVA supports had dissolved completely. Model then left to dry at room temperature for an additional 12 hours. | Washed and cured per FormLabs recommendations for material. Supports removed after processing. |

Supplemental material 1. Material printing profiles
