# Supplementary material for: Design, printing optimization, and material testing of a 3D-printed nasal osteotomy task trainer
Source: 3D Print Med. 2023 Jul 13;9:20. doi: 10.1186/s41205-023-00185-9 (PMC10339601; doi:10.1186/s41205-023-00185-9)
Supplement: Supplementary file 2 — Additional file 2: Material testing questionnaire for nasal osteotomy simulation. [file 41205_2023_185_MOESM2_ESM.pdf]

Nasal Osteotomy Material Test

1. Experience Level

   Mark only one oval.

   - Attending
   - Fellow
   - Resident

2. Have you completed over 20 independent nasal osteotomies?

   Mark only one oval.

   - Yes
   - No

3. First model type:

   Mark only one oval.

   - A
   - B
   - C
   - D
   - E

4. Model 1 - endonasal

Mark only one oval per row.

	Very Poor	Poor	Acceptable	Good	Very Good
**Tactile Feedback - (I.e. does the material feel like native bone, and respond to manipulation/instrumentation the way native bone would)**	⬭	⬭	⬭	⬭	⬭
**Audio Feedback**	⬭	⬭	⬭	⬭	⬭
**Ability to complete operative steps without limitation from material**	⬭	⬭	⬭	⬭	⬭

5. Model 1 - lateral percutaneous

Mark only one oval per row.

	Very Poor	Poor	Acceptable	Good	Very Good
**Tactile Feedback - (I.e. does the material feel like native bone, and respond to manipulation/instrumentation the way native bone would)**	⬭	⬭	⬭	⬭	⬭
**Audio Feedback**	⬭	⬭	⬭	⬭	⬭
**Ability to complete operative steps without limitation from material**	⬭	⬭	⬭	⬭	⬭

6. Model 1 - Overall

Mark only one oval per row.

	Very Poor	Poor	Acceptable	Good	Very Good
**Overall value as a learning aid**	⬭	⬭	⬭	⬭	⬭
**Reality of the model**	⬭	⬭	⬭	⬭	⬭

Model 2

7. Second model type:

Mark only one oval.

- ( ) A
- ( ) B
- ( ) C
- ( ) D
- ( ) E

8. Model 2 - endonasal

Mark only one oval per row.

	Very Poor	Poor	Acceptable	Good	Very Good
**Tactile Feedback - (I.e. does the material feel like native bone, and respond to manipulation/instrumentation the way native bone would)**	( )	( )	( )	( )	( )
**Audio Feedback**	( )	( )	( )	( )	( )
**Ability to complete operative steps without limitation from material**	( )	( )	( )	( )	( )

9. Model 2 - lateral percutaneous

Mark only one oval per row.

	Very Poor	Poor	Acceptable	Good	Very Good
**Tactile Feedback - (I.e. does the material feel like native bone, and respond to manipulation/instrumentation the way native bone would)**	⬭	⬭	⬭	⬭	⬭
**Audio Feedback**	⬭	⬭	⬭	⬭	⬭
**Ability to complete operative steps without limitation from material**	⬭	⬭	⬭	⬭	⬭

10. Model 2 - Overall

Mark only one oval per row.

	Very Poor	Poor	Acceptable	Good	Very Good
**Overall value as a learning aid**	⬭	⬭	⬭	⬭	⬭
**Reality of the model**	⬭	⬭	⬭	⬭	⬭

Model 3

11. Third model type:

Mark only one oval.

- ( ) A
- ( ) B
- ( ) C
- ( ) D
- ( ) E

12. Model 3 - endonasal

Mark only one oval per row.

	Very Poor	Poor	Acceptable	Good	Very Good
**Tactile Feedback - (I.e. does the material feel like native bone, and respond to manipulation/instrumentation the way native bone would)**	( )	( )	( )	( )	( )
**Audio Feedback**	( )	( )	( )	( )	( )
**Ability to complete operative steps without limitation from material**	( )	( )	( )	( )	( )

13. Model 3 - lateral percutaneous

Mark only one oval per row.

	Very Poor	Poor	Acceptable	Good	Very Good
**Tactile Feedback - (I.e. does the material feel like native bone, and respond to manipulation/instrumentation the way native bone would)**	⬭	⬭	⬭	⬭	⬭
**Audio Feedback**	⬭	⬭	⬭	⬭	⬭
**Ability to complete operative steps without limitation from material**	⬭	⬭	⬭	⬭	⬭

14. Model 3 - Overall

Mark only one oval per row.

	Very Poor	Poor	Acceptable	Good	Very Good
**Overall value as a learning aid**	⬭	⬭	⬭	⬭	⬭
**Reality of the model**	⬭	⬭	⬭	⬭	⬭

Model 4

15. Fourth model type:

Mark only one oval.

- A
- B
- C
- D
- E

16. Model 4 - endonasal

Mark only one oval per row.

	Very Poor	Poor	Acceptable	Good	Very Good
**Tactile Feedback - (I.e. does the material feel like native bone, and respond to manipulation/instrumentation the way native bone would)**					
**Audio Feedback**					
**Ability to complete operative steps without limitation from material**					

17. Model 4 - lateral percutaneous

Mark only one oval per row.

	Very Poor	Poor	Acceptable	Good	Very Good
**Tactile Feedback - (I.e. does the material feel like native bone, and respond to manipulation/instrumentation the way native bone would)**	⬭	⬭	⬭	⬭	⬭
**Audio Feedback**	⬭	⬭	⬭	⬭	⬭
**Ability to complete operative steps without limitation from material**	⬭	⬭	⬭	⬭	⬭

18. Model 4 - Overall

Mark only one oval per row.

	Very Poor	Poor	Acceptable	Good	Very Good
**Overall value as a learning aid**	⬭	⬭	⬭	⬭	⬭
**Reality of the model**	⬭	⬭	⬭	⬭	⬭

Model 5

19. Fifth model type:

Mark only one oval.

- A
- B
- C
- D
- E

20. Model 5 - endonasal

Mark only one oval per row.

	Very Poor	Poor	Acceptable	Good	Very Good
**Tactile Feedback - (I.e. does the material feel like native bone, and respond to manipulation/instrumentation the way native bone would)**					
**Audio Feedback**					
**Ability to complete operative steps without limitation from material**					

21. Model 5 - lateral percutaneous

Mark only one oval per row.

	Very Poor	Poor	Acceptable	Good	Very Good
**Tactile Feedback - (I.e. does the material feel like native bone, and respond to manipulation/instrumentation the way native bone would)**	◯	◯	◯	◯	◯
**Audio Feedback**	◯	◯	◯	◯	◯
**Ability to complete operative steps without limitation from material**	◯	◯	◯	◯	◯

22. Model 5 - Overall

Mark only one oval per row.

	Very Poor	Poor	Acceptable	Good	Very Good
**Overall value as a learning aid**	◯	◯	◯	◯	◯
**Reality of the model**	◯	◯	◯	◯	◯

Please rank the models in order of preference

23. First choice

Mark only one oval.

- A
- B
- C
- D
- E

24. Second choice

Mark only one oval.

- A
- B
- C
- D
- E

25. Third choice

Mark only one oval.

- A
- B
- C
- D
- E

26. Fourth choice

Mark only one oval.

- A
- B
- C
- D
- E

27. Fifth choice

Mark only one oval.

- A
- B
- C
- D
- E

This content is neither created nor endorsed by Google.

Google Forms
